# Research advances in the identification of regulatory mechanisms of surfactin production by *Bacillus*: a review

**DOI:** 10.1186/s12934-024-02372-7

**Published:** 2024-04-02

**Authors:** Junqing Qiao, Rainer Borriss, Kai Sun, Rongsheng Zhang, Xijun Chen, Youzhou Liu, Yongfeng Liu

**Affiliations:** 1 Institute of Plant Protection, Jiangsu Academy of Agricultural Sciences, Nanjing, 210014 Jiangsu China; 2 Institute of Biology, Humboldt University Berlin, Berlin, Germany; 3 College of Plant Protection, Yangzhou University, Yangzhou, 225009 Jiangsu China

**Keywords:** *Bacillus*, Surfactin, Regulatory mechanism

## Abstract

Surfactin is a cyclic hexalipopeptide compound, nonribosomal synthesized by representatives of the *Bacillus subtilis* species complex which includes *B. subtilis* group and its closely related species, such as *B. subtilis subsp subtilis, B. subtilis subsp spizizenii, B. subtilis subsp inaquosorum*, *B. atrophaeus, B. amyloliquefaciens, B. velezensis* (Steinke mSystems 6: e00057, 2021) It functions as a biosurfactant and signaling molecule and has antibacterial, antiviral, antitumor, and plant disease resistance properties. The *Bacillus* lipopeptides play an important role in agriculture, oil recovery, cosmetics, food processing and pharmaceuticals, but the natural yield of surfactin synthesized by *Bacillus* is low. This paper reviews the regulatory pathways and mechanisms that affect surfactin synthesis and release, highlighting the regulatory genes involved in the transcription of the *srfAA-AD* operon. The several ways to enhance surfactin production, such as governing expression of the genes involved in synthesis and regulation of surfactin synthesis and transport, removal of competitive pathways, optimization of media, and fermentation conditions were commented. This review will provide a theoretical platform for the systematic genetic modification of high-yielding strains of surfactin.

**Graphical Abstract:**

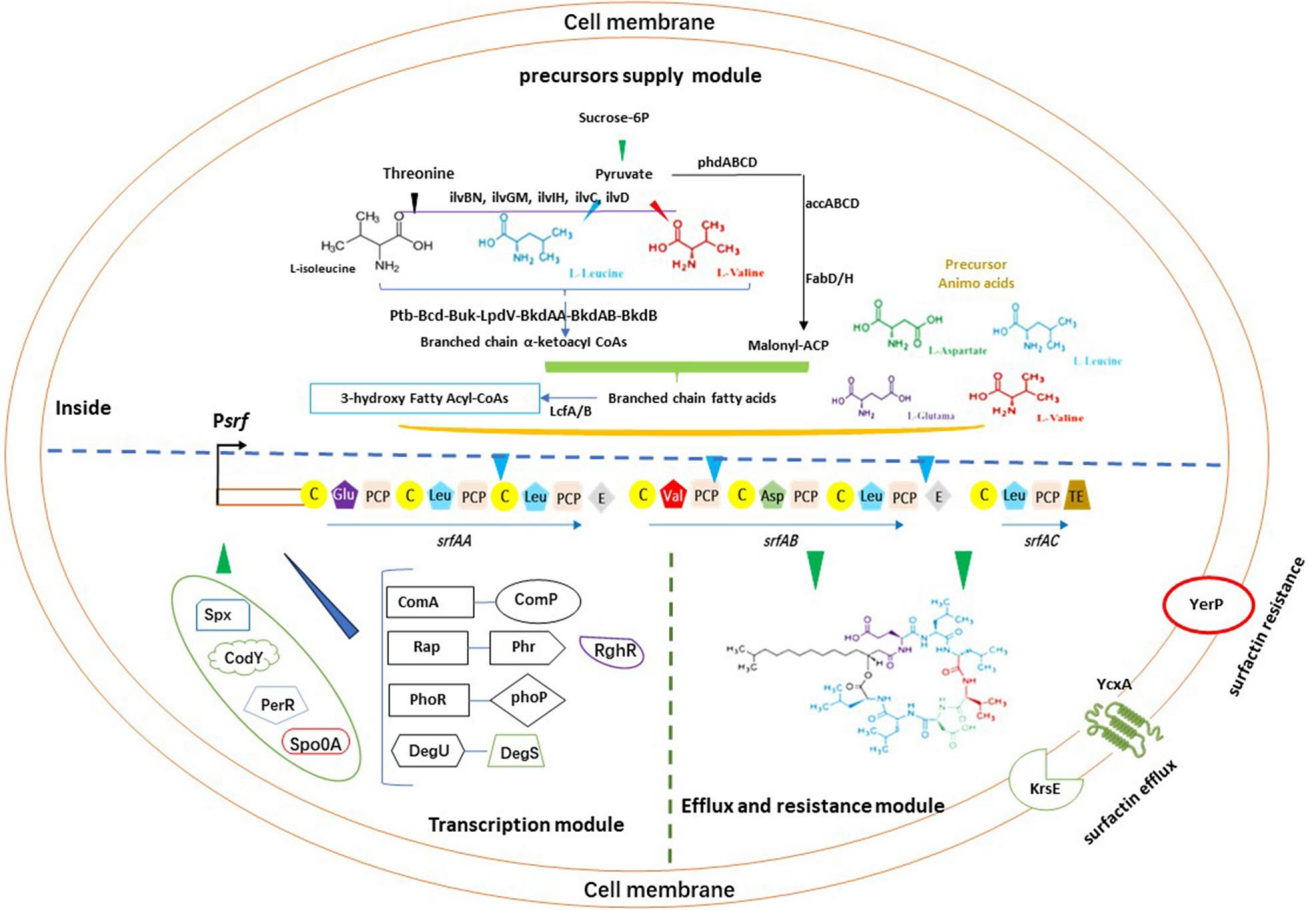

## Introduction

Recently, biopesticides made by antagonistic *Bacillus* species or their metabolites have been used to reduce the application of chemical pesticides in agriculture [1]. The most used *Bacillus* species include *Bacillus subtilis, Bacillus velezensis,* and *Bacillus thuringiensis*, such as *B. velezensis* FZB42, *B. subtilis* Bs916, *B. subtilis* QST713, and *B. thuringiensis* HD1, which were used in rice, wheat, maize, cotton, tomato, lettuce and cucumber [2–5]. The biocontrol efficiency of *Bacillus* strain relies on three main traits: ecological fitness, strong antagonistic activity toward plant pathogens, and an ability to trigger plant immune reaction [6]. The cyclic lipopeptides (LPs) of the surfactin, iturin and fengycin families produced by *Bacillus* strains are the key biocontrol elements which contribute to the traits mentioned above, especially antagonistic activity of biocontrol agents [6–8]. Especially surfactin is a key element in plant-bacteria interactions, enabling the *Bacillus* cells to form biofilms on plant roots, and to stimulate induced systemic resistance in plants. Cross talks between plants and bacteria are accomplished by a bacterial sensing system for plant pectin leading to enhanced surfactin synthesis in *B. velezensis* [9]. In addition, surfactin is directly or indirectly involved in several processes of cell differentiation, such as development of competence, motility, matrix production, cannibalism, *quorum sensing* and endospore formation [10–12].

Surfactin is a representative biosurfactant of the cyclic lipopeptide family which is synthesized by nonribosomal peptide synthetases (NRPS) and then transported outside the cells. Its chemical structure consists of a cycloheptapeptide, which is interlinked with β-hydroxy fatty acid chains of variable length containing 12−17 carbon atoms. Giant biosynthetic gene clusters (BGCs) involved in synthesis of surfactin were detected in many representatives of the *Bacillus subtilis* species complex, such as *B. subtilis*, *B. atrophaeus*, *B. spizizenii*, *B. amyloliquefaciens*, and *B. velezensis* [1, 13]. The surfactin BGC (e. g. BGC0000433, *B. velezensis* FZB42*,* [14] contains a co-encoded regulatory gene *comS*, which is located within the open reading frame of the *srfAB* gene [15]. ComS is involved in regulation of genetic competence [16], and simultaneously part of the *quorum sensing* system [17]. Natural variants of the surfactant surfactin are lichenysin from *B. licheniformis* [18] and pumilacidin from *B. pumilus* [19].

Due to its complex unique structure, surfactin can reduce the surface tension of water from 72 to 27.90 mN /m and possesses high thermal stability and salt resistance. Surfactin is also among the most well-known lipopeptide antibiotics with broad-spectrum antibacterial, antiviral and antitumor properties. Surfactin has great potential applications in agriculture, oil recovery, cosmetics, food processing and pharmaceuticals because of its structural stability, surfactant and antibiotic activity [20–22]. However, surfactin commercial application has been hindered due to the low yield obtained from *Bacillus* cultures. Therefore, several investigations have been focused on identification of the key regulatory mechanisms of surfactin biosynthesis to enhance surfactin production.

This review outlines a general overview of the regulation mechanisms affecting surfactin biosynthesis, and comments the attempts to enhance surfactin production. Starting with the regulation of transcription of the *srfAA-AD* operon, we also review the mechanisms of the export of surfactin from the cells. Special attention is given to the genes involved in synthesis of the necessary precursors for surfactin synthesis, such as branched-chain fatty acids, and amino acids, which are available only in limited amounts in the producer cells. Other factors, suitable to enhance yield of surfactin, such as removal of competitive metabolic pathways, and optimization of media and fermentation conditions were also considered. This review is aimed to provide a knowledge base for applying systematic genetic and other strategies for enhancing surfactin production, and for generating novel surfactin variants.

## Genes affecting surfactin expression

To increase the production of surfactin, several scientists have investigated the regulatory pathways and mechanisms that affect its synthesis and release. On the transcriptional level, expression of the surfactin biosynthesis pathway is—besides their general control—mainly influenced by five features: (1) the supply of β-hydroxy fatty acids; (2) the biosynthesis of α-amino acids; (3) the assembly of fatty-acid chains and amino acids, in which amino acids are sequentially assembled onto fatty acyl-coenzyme A through the NRPS system; (4) the release of surfactin from the *Bacillus* cells; (5) removal of other gene clusters involved in competitive synthesis pathways. The mechanism of secretion of surfactin is not fully understood, and excess surfactin can be toxic to *Bacillus* cells [23]. The gene categories mentioned above directly or indirectly regulate the synthesis and release of surfactin. The specific regulatory genes are listed in Table 1

**Table 1:** Summary of regulators involved in the synthesis and efflux of surfactin in *Bacillus*

Regulation pathway	Regulator	Function	Effect on surfactin yield	References
Transcription of the *srfAA-AD* operon	ComX, ComA-ComP	ComP responds to the extracellular peptide pheromone ComX, then undergoes autophosphorylation and subsequently interacts with ComA to form ComA -P, ComA-P activates transcription of the *srfAA-AD*	Positive	[24–26]
	RapC, RapF, RapA4	Binding with ComA, then inhibits ComA-P and Psrf interaction	Negative	[27–32]
	RapD, RapG, RapH, RapK	Overexpression of these Rap proteins inhibits *srfAA-AD* expression	Negative	[33–36]
	Rap60, RapQ	Regulates ComA activity by forming a ternary complex with ComA and DNA and inhibit *srfAA-AD* expression	Negative	[37–39]
	PhrC, PhrF, PhrG, PhrH, PhrK, Phr60, PhrQ, PhrA4	Inhibits the activity of their cognate Rap proteins	Positive	[27–30, 33–35, 37–39]
	RsiX, SigX	Disruption of *rsiX* activates *sigX*, which increases *rapD* expression	Negative	[40]
	RghR	Repress *rapD*, *rapG*, and *rapH* expression	Positive	[35]
	PhoR-PhoP	Positively regulates surfactin production under low phosphorus conditions	Positive	[41–44]
	DegU	DegU directly binds to the *srfAA-AD* promoter or indirectly regulates *srfAA-AD* expression by regulating other genes in undomesticated wild strain. Knock out of the *degU* gene enhances surfactin production	Negative	[45–48]
	Spo0A	Global regulator initiates sporulation. Deletion of *spo0A* enhances surfactin synthesis	Negative	[49]
	CodY	A global regulator that inhibits *srfAA-AD* transcription by competing with RNA polymerase binding sites in the *srfAA-AD* promoter region	Negative	[50]
	Spx	Spx occupies overlapping sites in the αCTD region of RNA polymerase which inhibits the binding between ComA-P and RNA polymerase with the *srfAA-AD* promoter	Negative	[51, 52]
	PerR	Competitively binding to the *srfAA-AD* promoter region bound by ComA-P	Negative	[53]
Branched chain fatty acid synthesis	AccA, AccB, AccC, AccD	Acetyl-CoA carboxylase (ACCase) complex, catalyzes the formation of malonyl-CoA from acetyl-CoA	Positive	[54]
	YngH	ACCase subunit (biotin carboxylase II), could maintain Acetyl-CoA ACCase activity	Positive	[55]
	FabD	Acyl carrier protein transacylase, converts Malonyl-CoA to malonyl-acyl carrier protein (ACP)	Positive	[56]
	FabHB	β-ketoacyl-acyl carrier protein synthase III (FabHB) catalyzes the condensation of β-keto acyl-ACP from malonyl-ACP and branched α-ketoacyl CoA	Positive	[56]
	LcfA, YhfL, YhfT, YngI	Fatty acyl-CoA ligases	Positive	[57–60]
Amino acid synthesis	YrpC, RacE, MurC	Associated with l-Glu consumption	Negative	[61, 62]
	PyrB, PyrC	Participates in the branching pathway for l-Asp biosynthesis, catalyzes the formation of Uracil from l-Asp	Positive	[61]
	bkdAA, bkdAB, lpdV	Involved in the l-Leu and l-Val metabolic pathways, alters the proportion of surfactin isoforms	Negative	[63, 64]
Surfactin secretion and immunity	YcxA, KrsE	Transport membrane proteins, YcxA and KrsE interact with the polar amino acid of surfactin	Positive	[65, 66]
	YerP	Transport membrane protein and homologous to the resistance and cytokinesis family of PMF-dependent efflux pumps	Positive	[23]

### A multitude of regulators affect transcription of the* srfAA-AD* operon

**Fig. 1:**
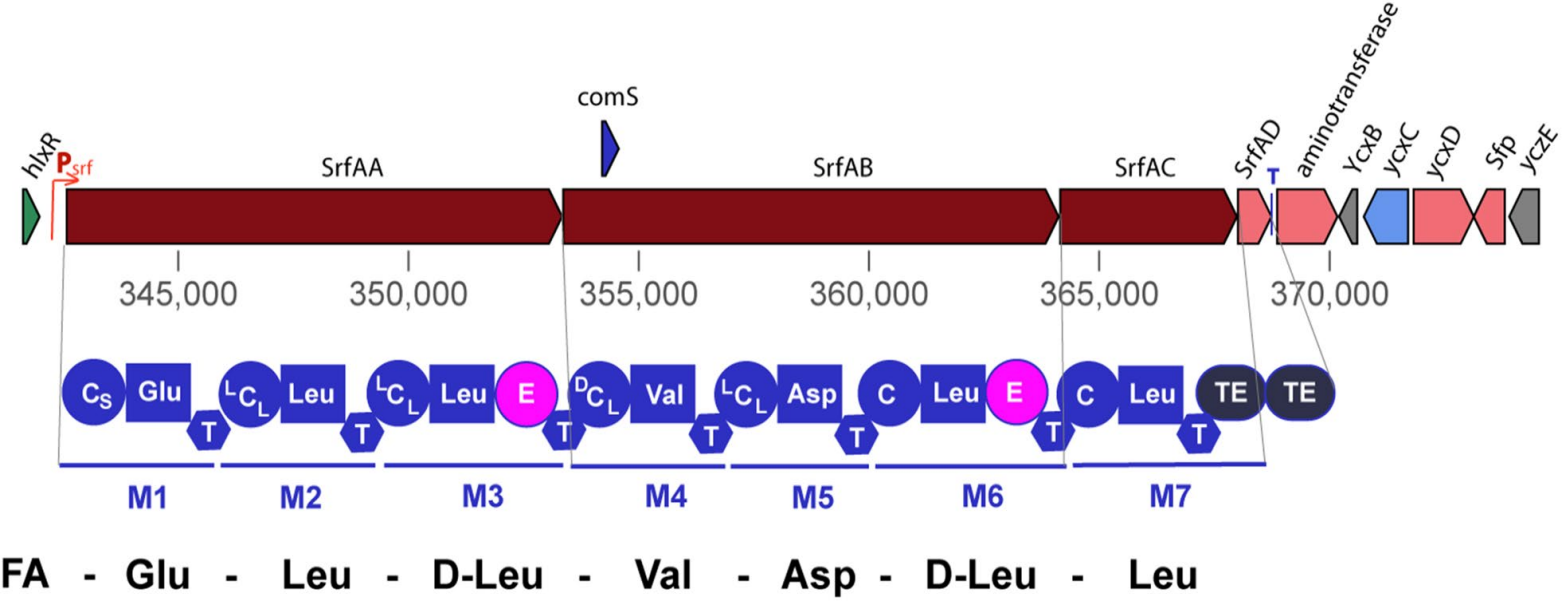
The surfactin gene cluster (BGC0000433) in *B. velezensis* FZB42. Transcription of the *srfAA-AD* operon is governed by the P_srf_ promoter. The *comS* gene is embedded within the *srfAB* gene, and is also transcribed by the P_srf_ promoter. Three genes, *srfAA*, *srfAB*, and *srfAC*, transcribe the nonribosomal peptide synthetases (NRPS) SrfAA, SrfAB, SrfAC, and the thioesterase SrfAD. SrfAA contains N-terminally the CS-domain and acylates the first amino acid Glu1 with various fatty acids. The elongation modules of SrfAA, SrfAB, and SrfAC yield the linear heptapeptide indicated at the bottom of the figure. The TE domain in SrfAC releases the lipopeptide and performs the cyclization between Leu7, and the fatty acid chain linked with Glu1. The second TE domain present in SrfAD seems to have mainly repair functions. The *sfp* gene, located downstream from the *srfAA-AD* operon encodes a phosphopantetheinyl transferase (PPTase), which is indispensable for nonribosomal synthesis of surfactin, the other lipopeptides (fengycin and bacillomycin D), and polyketides in FZB42. The *yczE* gene product, a membrane protein with unknown function, was also shown to be essential for synthesis of cyclic lipopeptides

The *srfAA-AD* operon transcribes the nonribosomal peptide synthetases and a thioesterase involved in surfactin synthesis (Fig. 1). The NRPS system consists of seven catalytic modules encoded by the genes *srfAA*, *srfAB*, *srfAC*, which is 27 kb in length [67, 68]. Each catalytic module is responsible for the recognition and condensation of an amino acid, and each module contains several catalytic structural domains: an adenylation (a) structural domain; a peptidyl carrier protein or thiolation (PCP) structural domain; a condensation (C) structural domain; a differential isomerization (E) domain; and a thioesterase (TE) structural domain, which is only present in the termination module [69–71]. Module 1 is responsible for the condensation of l-Glu; modules 2, 3, 6, and 7 are responsible for the condensation of l-Leu; module 4 is responsible for the condensation of l-Val; and module 5 is responsible for the condensation of l-Asp. *srfAD* is a type II thioesterase gene responsible for the cyclization and release of surfactin [72]. Downstream to the *srfAA-AD* operon, there is a phosphopantothionine ethantransferase (PPTases) gene, encoding the Sfp protein that activates surfactin synthesis [73]. Deletion of the *sfp* gene results in the inability to synthesize all three classes of lipopeptide antibiotics in *Bacillus* [74].

**Fig. 2:**
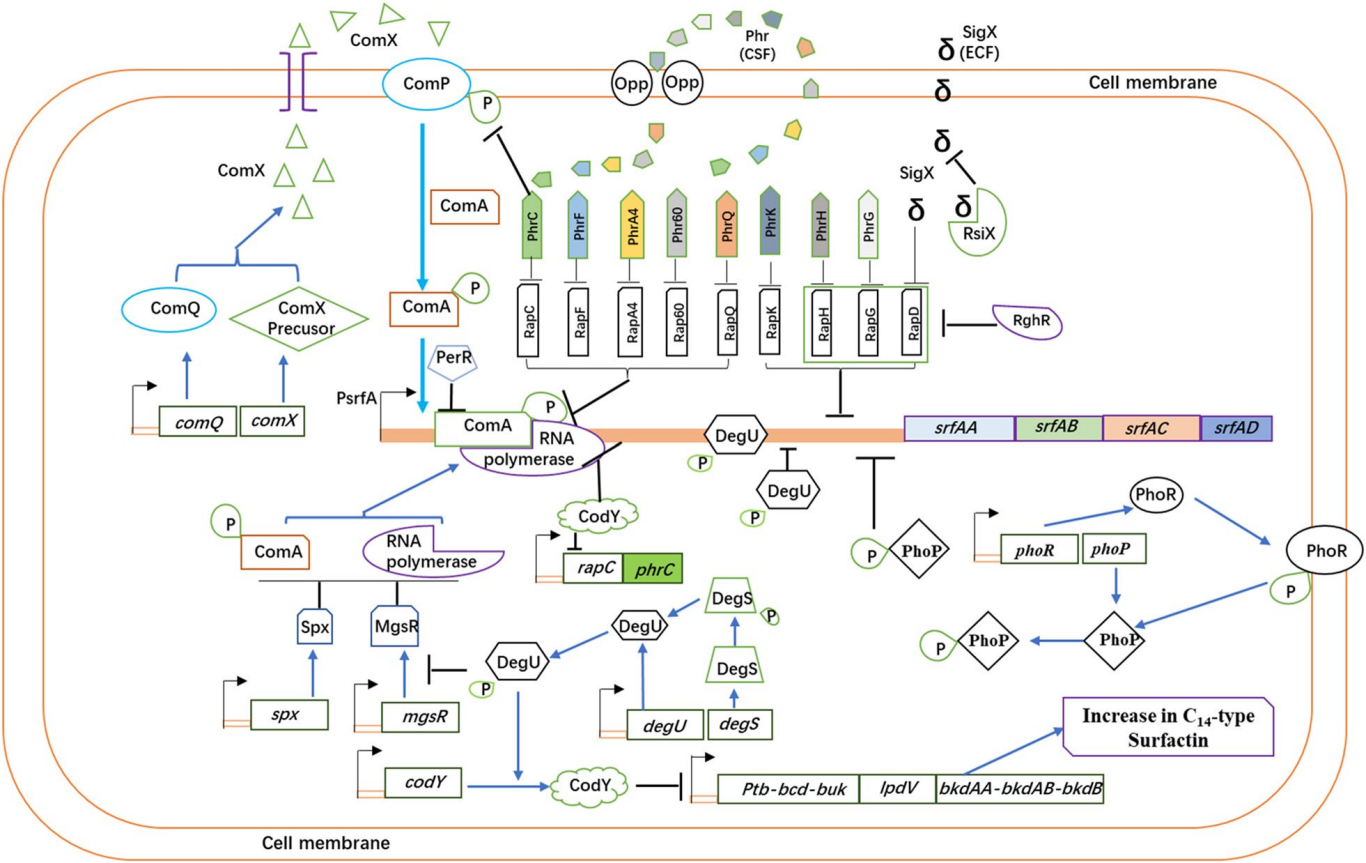
Schematic model for the regulation pathway of transcription of the *srfAA-AD* cluster. Black bent arrow represents the promoter of gene or operon. Black T-bar indicates the negative effects on DNA binding or protein interactions. ‘P’ in the circle means the phosphoryl group

The *srfAA-AD* operon transcription is governed by the SigA-dependent promoter P_srf_. Studies have shown that *srfAA-AD* expression is influenced by several regulatory factors and pathways (Fig. 2). Transcription of the *srfAA-AD* operon is activated by binding of phosphorylated ComA within the surfactin promoter region upstream of the *srfAA* gene. Further transcription factors, positively affecting transcription of the *srfAA-AD* operon are PerR and PhoP, whilst Abh, CodY, and Spx have a negative effect, and can be removed in order to enhance transcription of the surfactin operon.

#### The ComA-ComP two-component system

The ComA-ComP two-component system is the primary regulatory system governing transcription of the *srfAA-AD* operon. The synthesis of surfactin starts with the production of ComX, an extracellular peptide pheromone that is continuously synthesized, and accumulated during cell growth. When the cells reach a certain density, ComX is sensed, and undergoes autophosphorylation by the histidine kinase ComP, and subsequently interacts with ComA to phosphorylate ComA (ComA-P). ComA-P then activates the *srfAA-AD* operon transcription by binding at two binary symmetric regions upstream of the P_srf_ promoter-35 sequence, and is therefore essential for transcription of the *srfAA-AD* operon [24–26].

#### The Rap-Phr two-component system

The Rap-Phr system, consisting of aspartate phosphatases (Rap) and their inhibitory oligopeptides (Phr), governs fine tuning of the ComA-P dependent transcription of the surfactin operon during cell growth [36]. Six Rap proteins (C, D, F, G, H, and K) are involved. RapC and RapF suppress *srfAA-AD* expression by binding with ComA, which competes with the ComA phosphorylation exerted by ComP [31, 32]. RapD, RapG, RapH, and RapK overexpression also leads to inhibition of the *srfAA-AD* transcription. Site-directed mutagenesis has shown that *rapG* disruption increases *srfAA-AD* expression at least twofold, whilst rapH disruption has little effect on *srfAA-AD* expression [33, 35, 36].

Rap protein activity is inhibited by the Phr peptides. For survival in hostile environments, *Bacillus* species utilizes the Rap-Phr two component system to govern the differentiation of its populations, where the Phr pentapeptides function as *quorum sensing* signals [75, 76]. The *phr* gene is activated during the transition phase between exponential growth and stationary phase to express a precursor peptide with a putative signal peptide. After export of the pre-Phr from the cell via the SecA secretion system, the signal peptide is cleaved off, and the active Phr pentapeptide is generated [77]. The Phr peptides are imported into the cell by the oligopeptide permease (Opp) system, and act as *quorum sensing* signal, when the population density reached a high level. Then, inside the cell, they inhibit Rap protein activity [78, 79]. *B. subtilis* encodes five Phr peptides (PhrC, PhrF, PhrG, PhrH, and PhrK) that inhibit the activity of their cognate Rap proteins, and so enable the *Bacillus* cells to overcome the Rap-dependent inhibition of the surfactin expression [34, 35]. Studies have demonstrated that the extracellular signaling molecule PhrC enters the cell and binds to the RapC protein, which inhibits the interaction between RapC and ComA-P and promotes the subsequent transcription of the *srfAA-AD* gene [28, 30]. Jung et al. [27] successfully increased *srfAA-AD* gene transcription and surfactin production by overexpressing the *comX* and *phrC* genes in *B. subtilis* pHT43. Liang et al. [29] demonstrated that a novel RapA4-PhrA4 system of *B. amyloliquefaciens* NAU-B3 regulates surfactin production similar to RapC-PhrC. RapD lacks the homologous Phr peptide. As such, RapD is positively regulated by the extracellular function of the σ-factor SigX, whereas SigX is negatively regulated by its cognate anti-σ factor RsiX. The disruption of *rsiX* results in the accumulation of SigX, which increases RapD production. Elevated levels of RapD downregulate the *srfAA-AD* expression in *B. subtilis* cells [40]. Furthermore, RghR can repress *rapD*, *rapG*, and *rapH* expression in *B. subtilis* cells. RghR indirectly increases *srfAA-AD* transcription by specifically binding to the promoter sequences of *rapD*, *rapG*, and *rapH*, which suppresses *rapD*, *rapG*, and *rapH* expression [33, 35].

The Rap60-Phr60 system found in the *B. subtilis* endogenous plasmid pTA1060 regulates surfactin expression. Rap60 regulates ComA activity in a manner that is unique to *B. subtilis* 168 by forming a ternary complex with ComA and DNA. The resulting complex inhibits ComA activity without interfering with DNA binding. In comparison, reactions involving RapC demonstrate alterations in DNA binding [37]. Phr60 is an important inhibitor of Rap60 activity. It was also demonstrated that the RapQ-PhrQ system of the endogenous cryptic plasmid pBSG3 in *B. amyloliquefaciens* B3 regulates surfactin production, competence and sporulation similar to Rap60–Phr60 system [38, 39].

#### The PhoR-PhoP two-component system

The PhoR-PhoP two-component system positively regulates surfactin production under phosphate limitation [44]. Dong et al. [43] demonstrated that *srfAA* gene expression was reduced in PhoR and PhoP mutants in low phosphorus conditions. Wild-type strain NCD-2 produced 2.3–6.4 times more surfactin than the PhoR and PhoP mutant strains. In a low phosphorus environment, the histidine protein kinase PhoR autophosphorylates PhoR-P, which is located on the cell membrane. It then transfers a phosphate group to the response regulator PhoP, located in the cytoplasm. PhoP-P regulates target gene expression by binding to the promoter region of the downstream target gene [41, 42].

#### The DegU-DegS two-component system

The two-component regulatory system DegU-DegS is involved in the expression of extracellular protease, and lipopeptide antibiotics during late growth phase [80]. DegU owning typical helix-turn-helix DNA sequence-binding structures, has an ability to regulate the gene transcription by binding to the promoter regions through phosphorylated DegU (DegU-P) or unphosphorylated DegU [81]. DegU regulates bacillomycin D biosynthesis positively, and surfactin biosynthesis negatively [45–48]. In 2016, Mathieu et al. [46] reported that the DegU-DegS system differentially regulates the “K-state” which is a growth-arrested state of cells induced by ComK regulon [82] in undomesticated wild and laboratory domesticated *B. subtilis* model strains. This, in turn, affects the efficiency of competence cell formation. The specific regulatory mechanisms associated with this interaction are as follows: a site-specific mutation in the *degQ* promoter of domesticated laboratory strain reduces *degQ* expression capacity. Lower *degQ* expression lowers DegU-P concentration within the cell; however, this has little or no effect on the transcription of the surfactin operon. Moreover, the ComS levels remain stable, which, in turn, increases the levels of the receptor transcriptional regulator ComK. Bacteria are more likely to enter the K-state, and to form receptor cells. Unmodified wild-type *Bacillus* cells have no point mutation in the *degQ* promoter. As such, these cells have high intracellular concentrations of DegU-P, which inhibit *srfAA-AD* operon transcription. Deletion of the *degQ* gene in *B. subtilis* led to a threefold increase in surfactin production [83]. These cells also have low levels of ComS, which decrease ComK levels. The bacteria are then less likely to enter the K-state, and less able to form competent cells. Research has also shown that unphosphorylated DegU can activate the transcription of ComK. Our recent study showed that *degU* mutation resulted in a significant increase of surfactin and decrease of fengycin in wild-type strain *B. subtilis* Bs916. It is possible that DegU directly binds to the *srfAA* promoter region or indirectly regulates *srfAA-AD* expression by regulating CodY, PhrC, and MgsR (unpublished).

#### The Spx, CodY, PerR and Spo0A proteins

The Spx, CodY and PerR regulator proteins in *B. subtilis* hinder the *srfAA-AD* promoter transcription. Spx prevents the ComA-P dependent *srfAA-AD* promoter transcription by occupying overlapping sites in the αCTD region of RNA polymerase. This inhibits the interaction between ComA-P and RNA polymerase on the *srfAA* promoter, which inhibits the expression of the *srfAA-AD* operon [51, 52]. PerR represses also transcription by competitive binding on the ComA-P binding site at the *srfAA* promoter region [53]. When the *spx* and *perR* genes in *B. subtilis* are suppressed, *srfAA-AD* transcription levels increase by 4.5–4.2-fold, respectively [56].

The CodY protein in *B. subtilis* is a global regulator that inhibits *srfAA-AD* transcription by competing with the RNA polymerase binding sites in the *srfAA* promoter region [50, 84]; High amino acid concentrations activate CodY, and enable its binding within the *srfAA* promoter region. Knockout of the *codY* gene in *B. subtilis* 168 increases surfactin production by approximately tenfold [62]. CodY also represses the transcription of the *bkd* gene cluster that is involved in branched-chain ketoacid and fatty acid biosynthesis [85], deletion of *codY* also results in changes of surfactin isoforms.

Phosphorylation of the master regulator Spo0A initiates the sporulation process by inhibiting AbrB leading to transcription of competence and sporulation factor CSF, and was shown to negatively control surfactin synthesis. Knock out of the *spo0A* gene enhances surfactin synthesis [49].

### Genes associated with branched-chain fatty acid synthesis in surfactin

Fatty acids are key structural elements in surfactin. As such, fatty acid biosynthesis, particularly of branched-chain fatty acids, is essential for surfactin synthesis [63, 64]. A large number of intermediates are involved in this biosynthetic pathway. Researchers have demonstrated that surfactin production is dependent on the regulation of certain intermediates.

#### Genes associated with Malonyl-coenzyme-A synthesis

The acetyl-CoA carboxylase (ACCase) enzyme complex is encoded by four genes (*accA*, *accB*, *accC*, and *accD*). The complex contains two key catalytic structural domains: a biotin carboxylase encoded by *accC*, and a carboxyltransferase encoded by *accA* and *accD*. Furthermore, *accB* participates in the reaction by encoding the biotin carboxyl carrier protein that attaches to the cofactor biotin [54]. *B. subtilis* catalyzes the formation of malonyl-CoA from acetyl-CoA through ACCase. This is the first and rate-limiting step in fatty acid synthesis. Wang et al. [55] demonstrated that an *yngH*-encoded ACCase subunit (biotin carboxylase II) could maintain acetyl-CoA ACCase activity. Inhibition of this particular ACCase subunit resulted in significantly greater decreases in ACCase activity and surfactin production. In contrast, overexpression of *yngH* in *B. subtilis* TS1726 significantly increased ACCase activity, and surfactin production increased by 43%. In addition, when *accBC* and *accAD* expression are blocked with antisense RNA, there is a small decrease in ACCase activity and surfactin production, respectively.

#### Genes associated with malonyl-acyl carrier protein (ACP) synthesis

Malonyl-CoA is converted to malonyl-ACP by acyl carrier protein transacylase (FabD). In *B. subtilis* 168, overexpression of *accABCD* and *fabD* increased surfactin production slightly by 14% [56].

#### Genes associated with β-keto acyl-ACP synthesis

The β-ketoacyl-acyl carrier protein synthase III (fabHB) catalyzes the condensation of β-keto acyl-ACP from malonyl-ACP and branched α-ketoacyl CoA. This reaction is the first step in branched-chain fatty acid biosynthesis. Wu et al. [56] enhanced branched-chain fatty acid synthesis by overexpressing *fabHB* in *B. subtilis* 168, which resulted in a significant increase in surfactin production.

#### Genes associated with 3-hydroxy fatty acyl-CoA synthesis

The final substrate involved in assembly of the surfactin fatty acid chain is 3-hydroxy fatty acyl-CoA. This molecule requires one of four fatty acyl-CoA ligases (LcfA, YhfL, YhfT, or YngI) [59, 60]. When LcfA or YhfL are involved in the reaction, CoA thioesters are formed from the combination of 3-hydroxy fatty acids and CoA. These thioesters are recognized by the donor site of the C-structural domain of the first module, which catalyzes the nucleophilic attack of the α-amino group of the PCP-bound glutamate in the thioester bond of the fatty acid. The resulting acylated glutamate is accepted by the next C-structural domain, which allows peptide assembly to continue. Once complete, the final molecule is released [57, 58]. In comparison, when YhfT is involved in the reaction, only acyl adenylate intermediates can be observed, and no CoA thioesters are formed. Instead, YhfT plays a role in surfactin production by transferring acyl-adenosine monophosphate (AMP) derivatives to ACP, which, in turn, transfers intermediates to the surfactin assembly line. Meanwhile, the role of YngI in surfactin production in *B. subtilis* requires further elucidation.

Kraas et al. [57] demonstrated that the knockout of *lcfA*, *yhfL*, *yhfT* or *yngI* in *B. subtilis* OKB105 reduced surfactin production by 38–55%. After knock out of all four genes surfactin production was found reduced by 84%. Since the complete deletion did not completely eliminate surfactin production, it can be assumed that other pathways provide fatty acids for surfactin production. This also shows that branched-chain fatty acid biosynthesis plays a significant role in surfactin biosynthesis.

### Genes involved in biosynthesis of the amino acids used in surfactin synthesis

Besides fatty acids, amino acids are important precursors for surfactin biosynthesis. Increasing the amount of available amino acid precursors increases surfactin production. In addition, it was shown that the production of surfactin could be regulated by affecting the pathway of biosynthesis of four amino acids (l-Glu, l-Leu, l-Val and l-Asp). One study found that enhancing the leucine metabolic pathway resulted in a 20.9-fold increase in surfactin production [62]. Wang et al. [61] demonstrated that *B. subtilis* 168 increased surfactin production when genes *yrpC*, *racE*, or *murC*, which are associated with l-Glu consumption in the branching pathway, were repressed. In contrast, when genes *pyrB* or *pyrC*, which participate in the branching pathway for l-Asp biosynthesis, were suppressed, surfactin production was reduced.

The genes *bkdAA* and *bkdAB* are involved in pathways leading to consumption of l-Leu and l-Val. Suppression of *bkdAA* or *bkdAB* increases surfactin production and alters the proportion of final surfactin isoforms; For example, C_14_-type surfactin increases by almost 60% [63]. BkdAA and BkdAB initiate these reactions by increasing the accumulation of l-Leu and l-Val, and blocking the *bkd* operon (*lpdV*, *bkdAA*, *bkdAB*, and *bkdB*) which resulted in the reducing of iso-C_13_ and iso-C_15_ fatty acid synthesis [64]. Furthermore, Dhali et al. [63] demonstrated that disruption of the dehydrogenase complex in *lpdV* mutants inhibited branched-chain amino acid utilization and CoA precursor conversion into their respective branched-chain fatty acids. This resulted in a 2.5-fold increase in C_14_-type surfactin.

Branched-chain fatty acids and amino acids are the key structural elements of surfactin, and isomers with branched-chain fatty acid variants are the main components of surfactin variants, accounting for about 78% of the total. The branched-chain fatty acid synthesis precursors isobutyryl-CoA, isovaleryl-CoA and α-methylbutyryl-CoA originate from the branched-chain amino acids l-valine, l-leucine and l-isoleucine, respectively [21]. As described above, the biosynthesis of branched-chain fatty acids and amino acids significantly influenced the biosynthesis of surfactin. Therefore, it is possible to enhance the accumulation of branched fatty acid chains and amino acids for surfactin biosynthesis by modifying the metabolic pathways genetically, in order to improve the production of surfactin.

### Overexpression of surfactin efflux pump genes avoid adverse action of excess surfactin

The above mentioned factors directly or indirectly affect surfactin synthesis, but the rate of surfactin release and the strain self-resistance to surfactin are important factors because excess surfactin concentrations are toxic to *Bacillus* cells. It was reported that surfactin began to destroy the integrity of the liposome (simulated cell membrane) at 10 mg/L, when the concertation of surfactin increased to 500 mg/L, the liposome thoroughly disappeared [66]. This indicated that the intracellular concentration of surfactin in the living microbial cells could not be too high, or the cell membrane might be destroyed. Tsuge et al. [23] reported that the cell survival rate of *B. subtilis* 168 was decreased with the increasing of surfactin. The cell survival rate was only 50% when the surfactin concentration in the medium reached 100 mg/L. Known lipopeptide transport proteins are YcxA, KrsE, and YerP, that utilize proton motive force as an energy source. Surfactin efflux is a two-step process wherein (1) the polar amino acid of surfactin reacts with certain amino acid residues at the substrate binding sites of YcxA or KrsE, and (2) the lipid fraction facilitates membrane binding and permeation through the hydrophobic channels formed by KrsE [65]. Li et al. [66] found that surfactin transfer was impossible in *B. subtilis* THY-7 strains bearing YcxA mutation, whilst overexpression of the full-length YcxA increased surfactin secretion by 89%. Overexpression of KrsE also increased surfactin production by 52%. YerP is homologous to the resistance and cytokinesis family of PMF-dependent efflux pumps. YerP has been shown to be essential for surfactin resistance in *B. subtilis* [23]. When YerP was overexpressed, surfactin production increased by 145%. Li et al. [66] postulated that YerP-mediated surfactin resistance is essentially similar to the transmembrane transport of surfactin by YerP. Taken together, overexpression of the genes acting as surfactin efflux pumps is a way to avoid cell toxicity caused by excess surfactin.

## Approaches for enhancing surfactin production

Several studies have reported that the surfactin titer of wild-type *Bacillus* strains is limited to 100–600 mg/L [86]. Therefore, it seems difficult to achieve a break-through in the yield of surfactin only by traditional screening of wild strains, and by optimization of media and fermentation conditions. The systematic genetic modification, based on target directed changes in the complex regulatory network involved in surfactin expression, seems to be a promising strategy for constructing high-yielding surfactin producers. By combining systematic genetic modification with the use of novel and innovative fermentation methods, such as high-cell-density fermentation, a significant break-through of the surfactin yield can be achieved.

The probably most spectacular example for successful strain improvement based on metabolic engineering was achieved with the *B. subtilis* model strain 168, which is per se unable to synthesize surfactin due to a nonsense mutation in its *sfp* gene [56]. Therefore, the ability to produce surfactin was restored by integrating the wild-type *sfp*^+^ gene, resulting in a surfactin yield of 0.4 g/L. Then, by removing of competing genes involved in biofilm formation, and nonribosomal synthesis of peptides and polyketides a 3.3-fold increase in productivity was obtained. In a further step, cellular resistance to surfactin was enhanced by overexpressing genes associated with export and self-resistance, such as *swrC* (*yerP*) and the *liaIHGFSR* operon. This results in an 8.5-fold increase of the surfactin titer. Next, by increased supply of branched-chain fatty acids, the surfactin yield was enhanced to 8.5 g/L corresponding to an increase of 20.3-fold. In a final step, supply of the available fatty acid precursor acetyl-CoA was enhanced by redirecting it from cell growth to surfactin synthesis. The final surfactin titer reached 12.8 g/L corresponding to 42% of the theoretical yield calculated for the substrate sucrose.

Applying alternative strategies, such as promoter substitution, genome reduction and genome shuffling might contribute also to higher surfactin yields [87]. Here, we shortly summarize the different strategies for enhancing surfactin production.

### Enhancement of transcription of the surfactin operon

The regulatory network governing the ComA dependent transcription of the surfactin operon has been extensively discussed in Sect. A multitude of regulators affect transcription of the srfAA-AD operon. It is recommended to enhance the transcription of the *srfAA-AD* operon though overexpression of ComX, PhrC and ComA, and decreasing the amount of DegU, CodY, Spx, PerR, which leads to the accumulation of the nonribosomal peptide synthetases SrfAA, SrfAB, and SrfAC for surfactin biosynthesis and improves assembly of the peptide moiety. Furthermore, the knockdown of *degU* could significantly increase the production of surfactin, while the biosynthesis of iturin and fengycin was almost completely inhibited [45].

### Promoter engineering

Surfactin expression can be enhanced by replacing the original P_srf_ promoter with stronger promoters, such as P_xyl_, P_spac_, and P_g3_ [21]. Replacement of the natural P_srf_ promoter by the IPTG inducible P_spac_ promoter resulted in a tenfold increase of the surfactin yield [88]. By using the artificial P_g3_ promoter, the surfactin titer was enhanced from 0.55 g/L produced in the original *B. subtilis* THY-7–9.74 g/L produced in the engineered strain. 0.14 g surfactin were obtained per g sucrose [89].

### Increase of the supply of the building blocks for surfactin synthesis

It is suggested that the metabolic pathways of fatty acids and amino acids could be genetically modified to increase the supply of branched fatty acid chains and the amino acids l-glutamate, l-leucine, l-valine, and l-aspartate involved in the surfactin biosynthesis. For instance, the suppression of the expression of the *bkd* operon (*lpdV, bkdAA, bkdAB* and *bkdB* genes) not only increased the accumulation of l-Leu and l-Val, but also increased the iso-C_14_ fatty acid accumulation [61]. By strengthening the leucine metabolic pathway, surfactin production was enhanced by more than 20-fold [62].

### Enhanced export from the cell

Enhancing the efflux of surfactin avoids toxic effects to the cell due to high surfactin concentration, however the exact mechanisms of the transport of surfactin through the cell membrane are still not clear. It is assumed that transmembrane exporters dependent on proton motive force are involved. It was reported that the *yerP* gene is involved in surfactin self-resistance [23]. Overexpression of the putative surfactin transporters YcxA, KrsE, and SwrC (YerP) resulted in an enhanced secretion of surfactin [66].

### Combinatorial biosynthesis for generation of more efficient surfactin

As well as surfactin high-yielding engineered strains, combinatorial biosynthesis strategies can also be used to modify the structure of surfactin to improve its effect. Combinatorial biosynthesis plays an important role in the structural modification of lipopeptides. It alters the lipopeptide biosynthetic pathway purposefully to create predictable structural products that exhibit new functions or activities as expected by the investigator. The structural modifications of the surfactin are mainly peptide rings and hydrophobic fatty acid chains [90].

### Application of genome reduction and shuffling strategies

Different methods based on genomic rearrangement (“genome shuffling”) [91] and reduction of the genome size were applied to enhance surfactin production. Three rounds of genome shuffling via recursive protoplast fusion in *B. velezensis* resulted in a fourfold increase of productivity from 229.6 mg/L–908,15 mg/L [92].

A genome reduced *B. subtilis* strain in which 10% of the whole genome, including the fengycin and bacilysin gene clusters, was removed, was found superior in its growth parameters, but did not surpass the original strain in surfactin productivity [93].

### Process and media optimization

The patent filed by Kaneka Corp. (US7011969B2) claims that surfactin concentrations of up to 50 g/L can be reached after a long-term fermentation of 80 h, performed with *B. subtilis* and soybean flour as carbon source. Unfortunately, a detailed description of the process parameters, and strain properties were not given [94].

High cell density fermentation following a fed-batch protocol was used for efficient surfactin production with the nonsporulating *Bacillus subtilis* strain 3NA, in which a *sfp*^+^ gene has been introduced [49]. A cell density of 88 g/L accompanied with a surfactin titer of 26.5 g/L was reached after 38 h fermentation, impressively underlining the power of optimizing fermentation process parameters, together with the use of purposefully engineered high-yielding production strains.

## Summary and outlook

In recent years, many efforts have been devoted to unravel the complex network involved in surfactin expression and to optimize the production process for a “green” surfactin to an economically attractive level. Rapid advances in the application of efficient genetic engineering techniques for the development of high-yielding strains, together with the use of high-cell density and other innovative fermentation techniques, now enable the production of surfactin in a range of 20 g/L and above. The success story reviewed here could also promote the development of highly efficient production processes for the biosynthesis of other “green” compounds which can be used as environmentally friendly tools in sustainable agriculture and industry. Examples include fengycins and iturin-like compounds with antifungal properties, as well as other active ingredients that are useful in biological plant protection.

### Definitions of the key concepts

**Nonribosomal peptide synthetases (NRPSs),** a large multi-enzyme is composed of repeating enzyme domains with modular organization to activate and couple fatty acids to l-amino acids, l-amino acids to l-amino acids, and D-amino acids to l-amino acids in a particular order to generate linear or cyclic peptides.

**Competence,** a distinct DNA uptake phenotype of *Bacillus subtilis* which appears to be a cell survival strategy for either procuring new genetic information or obtaining DNA as food. The competence is correlated with high cell density and nutrient limiting conditions.

**“K-state”,** a growth-arrested state of *Bacillus* cells induced by transcription factor ComK, of which competence for genetic transformation is but one notable feature. This is a unique adaptation to stress and the persistent state has been defined the “K-state”.

**Combinatorial biosynthesis,** an approach to produce novel natural products with modifying of biosynthetic pathways by genetic engineering. The feasibility of this approach was demonstrated in biosynthesis of lipopeptide, polyketides and nucleoside antibiotics.

**Genome shuffling,** a method that combines DNA shuffling with the recombination of entire genomes which provide an alternative to the rapid production of improved strains in microorganisms metabolic engineering breeding.

## Data Availability

Not applicable.
